# Polysaccharides of *Dendrobium officinale* Kimura & Migo Leaves Protect Against Ethanol-Induced Gastric Mucosal Injury *via* the AMPK/mTOR Signaling Pathway *in Vitro* and *vivo*

**DOI:** 10.3389/fphar.2020.526349

**Published:** 2020-11-11

**Authors:** Yang Ke, Lianghui Zhan, Tingting Lu, Cong Zhou, Xue Chen, Yingjie Dong, Guiyuan Lv, Suhong Chen

**Affiliations:** ^1^Collaborative Innovation Center of Yangtze River Delta Region Green Pharmaceuticals, Zhejiang University of Technology, Hangzhou, China; ^2^College of Pharmaceutical Science, Zhejiang Chinese Medical University, Hangzhou, China

**Keywords:** *Dendrobium officinale* leaves (LDOP), polysaccharides, alcohol, gastric injury, autophagy

## Abstract

Ethanol-induced gastric mucosal injury is a common gastrointestinal disorder. Polysaccharides separated from herbs have been shown to be effective for ethanol-induced gastric mucosal injury, but whether the polysaccharides from *Dendrobium officinale* Kimura & Migo leaves (LDOP-1) protected mucosa from ethanol-induced injury remains unknown. Thus, the present study carried out gastric mucosal protection and the mechanism of LDOP-1 in *vivo* and *vitro*. The chemical composition of LDOP-1 was a heteropolysaccharide comprising mannose, galacturonic acid, glucose, galactose, and arabinose at a molar ratio of 2.0:1.1:0.7:0.5:0.4. Pharmacological results showed that LDOP-1 significantly reduced gastric mucosal injury score and pathological injury, improved antioxidant capacity, reduced the level of reactive oxygen species, and reversed the apoptosis of GES-1 in *vivo* and *vitro*. Research showed that LDOP-1 pretreatment upregulated the expression level of p-AMPK, LC3β, HO-1, and Beclin-1; downregulated the expression level of p-mTOR and p62; and reversed the expression level of caspase3, Bax, and Bcl-2. This study was the first to demonstrate that LDOP-1 could protect against ethanol-induced gastric mucosal injury via the AMPK/mTOR signaling pathway in *vitro* and *vivo*.

## Introduction

Ethanol-induced gastric mucosal injury is a common gastrointestinal disorder that is characterized by hemorrhage, erosion, ulcers, and loss of the gastric mucosa (Matsuda et al., 2002; Senol et al., 2011). In general, the defensive and repairing effects of ethanol-induced gastric mucosal injury are less noxious factors, and the gastric mucosal epithelial cells could be attacked (Goel et al., 2005). Excessive alcohol intake could induce gastric mucosal injury through stimulating mucosal epithelial cell apoptosis, inflammatory reaction, and oxidative stress in gastric tissue (Jiang et al., 2015). Recent research has shown that ethanol-induced damage to the gastric mucosa likely occurred as a result of oxidative stress because of excessive production of reactive oxygen species (ROS) during ethanol metabolism (Zeng et al., 2017). To prevent the generation of ROS, mammalian cells have developed many antioxidant defense molecules, such as total antioxidant capacity (T-AOC) and superoxide dismutase (SOD), which scavenged oxygen-derived free radicals directly or increased levels of radical scavengers (Yang et al., 2017). In addition, some reports have found that the generation of ROS could activate caspase and downregulate the ratio of Bcl-2 and Bax, which leads to cell apoptosis (Fulda et al., 1997; Wang et al., 2017). This finding indicates that the antioxidant system plays an important role in protecting the gastrointestinal mucosal layer against gastric damage (Shin et al., 2013).

Recently, extensive attention has been paid to the role of autophagy in many kinds of diseases (White et al., 2011; Singh et al., 2017). In addition, increasing evidence shows that moderate levels of autophagy could be an important cellular protective mechanism that contributes to cell survival and reduces oxidative damage and ROS levels by scavenging protein multimers and damaged organelles (Ureshino et al., 2014; Li et al., 2015). The three primary proteins that monitor the formation of autophagosomes and autolysosomes are LC3, Beclin-1, and p62 (Yang et al., 2018). Moreover, Beclin-1 and the lipidization of LC3 promote the formation of autophagosomes, and p62 inhibits the formation of autophagosomes (Hao et al., 2019). Furthermore, AMP-activated protein kinase (AMPK), acting as a metabolic checkpoint inhibiting cellular growth (Mihaylova and Shaw, 2011), may lead to autophagy through the negative regulation of rapamycin (mTOR) (Yu et al., 2017), which is one of the important molecules regulating autophagy (Hay and Sonenberg, 2004). Therefore, autophagy has emerged as a research focus and shown a potential therapeutic target to protect gastric mucosal injury from oxidative damage (Zhang et al., 2017; Zhai et al., 2018).

Several researchers have investigated the effects of certain polysaccharides separated from herbal medicines in the protection against ethanol-induced gastric mucosal injuries by increasing the antioxidant defense system (Qin et al., 2017). *Dendrobium officinale* Kimura & Migo is one of the traditional Chinese medicinal herbs, which has been used as an herbal medicine in many Asian countries for centuries because of its traditional function of nourishing the stomach (Xie et al., 2010). Based on previous reports, the polysaccharide component could decrease ulcer lesions and histological changes on the ethanol-induced gastric damage in rats and improve the activity of SOD and PGE_2_ (Zhang et al., 2018). However, whether the polysaccharide separated from *Dendrobium officinale* Kimura & Migo leaves (LDOP), as a new food resource, has a similar effect to the polysaccharide from *Dendrobium officinale* Kimura & Migo stems is still not clear. Based on our previous study, it has been found that LDOP had an anti-inflammatory effect on ethanol-induced gastric mucosal injury in rats. However, no report was found whether LDOP could protect gastric mucosal injury against ethanol with regard to antioxidant ability via the AMPK/mTOR signaling pathway.

In this study, we first investigated the potential gastrointestinal effects of LDOP-1 on the ethanol-induced gastric mucosal injury model from the perspective of oxidation resistance by activating the AMPK/mTOR signaling pathway in *vivo*. Then, we further studied its protective mechanism by adding AMPK-related activators in *vitro*.

## Method

### Chemicals and Reagents

*Dendrobium officinale* Kimura & Migo leaves were supplied by Senyu Holding Group (Zhejiang, China) which was identified by Herbarium Kunming Institute of Botany Chinese Academy of Sciences. The batch number of the sample used in this experiment was sy20180615 and sy20180618. Omeprazole (B1702400) was bought from Madaus GmbH (Beijing, China). Compound C was bought from ApexBio (Houston, America). AICAR (S1516) and rapamycin (S1842) were bought from Beyotime Biotechnology (Beijing China). Ammonium pyrrolidine dithiocarbamate (PDTC, S1808) and lactate dehydrogenase (LDH) assay kit (C0017) were obtained from Beyotime (Beijing, China). Biochemical assay kits of SOD (A001-3-2), T-AOC (A015-1-2), and ROS (E004-1-1) were purchased from Nanjing Jiancheng Bioengineering Institute (Nanjing, China). Polyclonal antibodies LC3β (Ab-AF4650) and p-mTOR (ET1610-93) were bought from Affinity Biosciences Ltd. (Changzhou, Jiangsu). Polyclonal antibodies of m-TOR (66888-1-1g), Beclin-1 (11306-1-AP), HO-1 (66743-1-Ig), Bax (50599-2-Ig), Bcl_2_ (60178-1-Ig), β-actin (66009-1-1g), HRP-conjugated affinipure goat anti-rabbit lgG(H+L) (SA00001-2), and HRP-conjugated affinipure goat anti-mouse lgG(H+L) (SA00001-1) were bought from Proteintech Group, Inc. (Wuhan, China). Alexa Fluor 350-labeled goat anti-mouse IgG(H+L) (A0412), Alexa Fluor 555-labeled donkey anti-rabbit IgG(H+L) (A0453), enhanced immunostaining permeabilization buffer (p0097), 4% paraformaldehyde fix solution, and 4% PFA fix solution (P0099) were bought from Beyotime (Beijing, China). Fetal bovine serum (FBS, 13011-8611) was bought from Zhejiang Tianhang Biotechnology Co., Ltd. (Zhenjiang, China). Trypsin-EDTA (9002-07-7) solution and Dulbecco’s modified eagle medium (12100) were obtained from Beijing Solarbio Science & Technology Co., Ltd. (Beijing, China).

### Preparation of LDOP-1

#### Preparation of LDOP Extract

One kilogram of dried *Dendrobium officinale* Kimura & Migo leaves was extracted by 5 L of distilled water three times for 2 h each extraction. Four liters of Ethanol was added to the extract four times after the combined extract (water extract with 3 times repetition) was concentrated to 1 L and placed in the fridge at 4 °C. Then, the precipitate was removed and placed in a vacuum drying oven. Finally, the LDOP was stored in the dryer in the form of powder. Then, LDOP was deproteinized, decolorized, and eluted with different solvents to obtain LDOP-1 (Zeng and Luo, 2007).

#### Content Detection of LDOP

Using D-glucose as the reference substance, the total sugar content was determined with the phenol-sulfuric acid method. The content of total flavonoid in *Thladiantha dubia* Bunge was determined by sodium nitrite-aluminum nitrate colorimetric with Rutin as the standard product and Polyphenol content was determined by the Folin–Ciocalteu assay using gallic acid as the reference substance (Zhao et al., 2016). The contents of total alkaloids were measured by acid dye colorimetry, and dendrobine served as the standard (Zeng and Luo, 2007). Using bovine serum albumin as the reference substance, the protein content was detected with the Coomassie brilliant blue method (Goldring, 2019).

#### Determination of LDOP Monosaccharide Composition by High-Performance Liquid Chromatography (HPLC)

In brief, LDOP acid was hydrolyzed by 0.5 M PMP (1-phenyl-3-methyl-5-pyrazolone) methanol solution at 70 °C for 100 min, and then the hydrochloric acid solution was added and mixed. Trichloromethane was extracted thrice, and the supernatant was obtained at 12 000 × g for 20 min. The derivative supernatant was analyzed using HPLC (Agilent Co., USA) with C18 column (5 μm, 4.6 mm × 250 mm, Agilent, USA) and detected using an ultraviolet detector at 250 nm. The HPLC analysis conditions were as follows: column temperature, 30 °C; mobile phase, 0.01% phosphate buffer and acetonitrile; flow rate, 1.0 mL min^−1^.

### Protective Effect of Polysaccharides on Gastric Mucosal Injury Rat Model

#### Animal and Treatment

Male Sprague-Dawley rats (200 ± 10 g, 2 months) were obtained from JOINN Laboratories (Suzhou) and raised in the animal room of Zhejiang University of Technology. The rats were given unlimited food and water with humidity of 50 %  ±  10% at 23 °C  ±  2 °C for a 12  h light/dark cycle. All experiments were performed in accordance with the Regulations of Experimental Animal Administration issued by the Ministry of Science and Technology of the People's Republic of China. This experiment was approved by the ethics committee of Zhejiang University of Technology.

All rats were divided into five groups randomly (*n* = 7): (1) control group, (2) model group, (3) LDOP-1-H (400 mg/kg LDOP-1), (4) LDOP-1-L (100 mg/kg LDOP-1), (5) OME (100 mg/kg Omeprazole). The rats from the control and model groups were given distilled water 1 mL/100 g weight, and the remaining groups were given the same volume/weight medicine for 30 days. On the last day, all rats were given orally with absolute alcohol (5 mL/kg) for 1 h after the last medication. Then, gastric tissues were collected followed by washing with cold saline, and blood was collected by the abdominal aortic method. After being sacrificed, all rats were stored in a −80 °C fridge. All experiments used 7 rats for testing.

#### Rat Gastric Injury Evaluation

After pylorus and door ligation, the stomachs were opened along the greater curvature, washed with cold saline, blotted dry using a filter paper, and pinned flat on a cardboard for gross lesion evaluation. The gastric ulcer index was determined on the basis of the Technical Specifications for Health Food Inspection and Evaluation (Ezer, 1988): spot erosion was recorded as 1 point; erosion length > 15 mm was recorded as 4 points; 10–15 mm was recorded as 3 points; 6–10 mm was recorded as 2 points; 1–5 mm was recorded as 2 points; erosion width > 2 mm was recorded as 2 points; 1–2 mm was recorded as 1 point. Moreover, the total score = spot point + length point + (width score × 2, **Table 1**).

**Table 1:** Gastric mucosal injury score.

Degree of damage	1 point	2 points	3 points	4 points
Spot	1	—	—	—
Length	1–5 mm	6–10 mm	10–15 mm	> 15 mm
Width	1–2 mm	> 2 mm		

Total score = spot point + length point + (width point × 2).

#### Biochemical Assays

After the damaged gastric tissue samples were fixed in 4% paraformaldehyde solution for 24 h, antrum tissue which was about 0.5 cm × 0.5 cm in size was cut from Gastric, and then dehydrated with 95% ethanol, embedded in paraffin. The embedded sections were cut using the microtome at a thickness of 4 μm and undergone hematoxylin and eosin (H&E) staining (Guo et al., 2016), PAS staining, which was used to analyze the mucin expression in the gastric mucosa (Raish et al., 2018), and semi-quantitative analysis of PAS was based on PMID: 23499292 and 23499292. Immunohistochemical staining with Bcl_2_, HO-1, and LC3B to evaluate the gastric mucosal damage and label the autophagosomes examined under a light microscope.

#### Histological Evaluations and Immunohistochemistry

The activity of SOD, T-AOC, ROS, and caspase-3 in serum or gastric tissue was measured by a biochemical kit according to the protocol provided by the manufacturer.

### Protective Effect of LDOP-1 on GES-1 Apoptosis

#### Cell Culture and Treatment

Gastric epithelial cell line (GES-1) was bought from Servicebio (Wuhan, China) and cultured in Roswell Park Memorial Institute 1640 supplemented with 10% FBS, 100 units/mL penicillin, and 100 μg/mL streptomycin; cell supernatant was changed every 2 days, and GES-1 was cultured at 5% CO_2_, 37 °C.

#### MTT Assay

GES-1 was seeded in 96-well plates at the density of 8 × 10^3^ cells/well. The cells were pretreated with serum-free medium, LDOP-1 (62.5, 125, 250 μg/mL), Compound C (6-[4-(2-Piperidin-1-ylethoxy) phenyl]-3-pyridin-4-ylpyrazolo [1,5-a] pyrimidine, 1 nM, the inhibitor of AMPK), AICAR (5-Aminoimidazole-4-carboxamide ribonucleotide, 0.5 mM, the activator of AMPK) for 12 h, and then exposed to 8% ethanol or medium for another 2 h. Then, the culture medium was replaced with 1 mg/mL MTT solution in a fresh medium and incubated for another 4 h. The supernatant was discarded, whereas the formazan was resolved in 100 μL DMSO. Optical density values were read at a wavelength of 570 nm by a microplate reader (BioTek Instruments, Inc., Beijing, China). The results were repeated three times.

#### Intracellular ROS Analysis

ROS in GES-1 was measured using a commercial ROS detection reagent through the DCFH-DA method according to the manufacturer’s instructions (Beyotime Jiangsu China). In brief, after being pretreated with LDOP-1 for 12 h, GES-1 were incubated with 8% ethanol for 3 h at 37 °C. Then, the cells were incubated with 10  µmol/L of DCFH-DA (2’,7’ –dichlorofluorescin) in the dark for 30 min at 37  °C and then washed with PBS. ROS generation was detected by using a spectrophotometer at a 488  nm excitation wavelength and 525  nm emission wavelength and a photographic microscope (Olympus Corporation, Tokyo, Japan).

#### Mitochondrial Membrane Potential Determination

Mitochondrial membrane potential was analyzed by the fluorescent dye JC-1. When the mitochondrial membrane potential is high, JC-1 accumulates in the matrix of the mitochondria to form a polymer, which produces red fluorescence. When the mitochondrial membrane potential is low, JC-1 cannot aggregate in the matrix of the mitochondria. As such, JC-1 is a monomer that produces green fluorescence. In general, GES-1 was labeled with JC-1 reagent (1 μg/mL) for 1 h at 37 °C in the dark. After washing, mitochondrial membrane potential was detected by using a microplate reader and a photographic microscope.

#### Fluorescence Microscopy

Firstly, GES-A cells were cultured at the density of 8×10^3^ cells/well at 5% CO_2_, 37 °C. GES-1 were washed twice with PBS and fixed with 4% formaldehyde for 15 min at room temperature. Then, cells were washed three times with wash buffer. Nonspecific binding sites were blocked for 60 min at room temperature with confining liquid. Then, without further washing, cells were incubated with Bcl-2, LC3β, and HO-1 antibodies. Afterward, the cells were diluted at 1:50 with goat serum and stored overnight at 4 °C. Bcl-2, LC3β, and HO-1 staining were revealed by incubating goat anti-mouse antibody or donkey anti-rabbit (1:500) for 60 min at room temperature. Then, GES-1 was incubated with FITC for 20 min.

#### LDH Cytotoxicity Assay

LDH release was determined using the LDH Cytotoxicity Assay Kit. In brief, GES-1 was cultured at the density of 8×10^3^ cells/well at 96-well plates and incubated with ethanol for 2 h after LDOP-1 or AICAR pretreatment for 12 h in RPMI-1640. Then, LDH was measured in the medium according to the manufacturer’s instruction.

#### Western Blot Assay

Western blot was used to detect LC3β, AMPK, m-TOR, Beclin-1, Heme Oxygenase 1, and Bax Bcl-2. The proteins were extracted with radio immunoprecipitation buffer, and the concentration was quantitated by BCA assay. Total protein (100 μg) was separated on 10% resolving SDS-PAGE gel and 5% stacking gel and transferred to a polyvinylidene difluoride membrane. Next, the membranes were blocked with 5% skim milk in Tris-buffered saline containing 0.1% Tween-20 (TBST) for 2 h, incubated with the primary antibody overnight at 4 °C, and then incubated with horseradish peroxidase-conjugated secondary antibody after washing with TBST. The membranes were visualized using an enhanced chemiluminescence detection system. The density of each band was estimated using the Image Lab software. All target proteins were normalized against the loading control β-actin.

### Statistical Analysis

Each experiment was performed at least three times, and the results were presented as means ± SD. The results were analyzed with IBM SPSS Statistics 19.0 (SPSS Inc., NY, USA). Significant differences were determined by Student’s t-test and one-way analysis of variance (*P* < 0.05 or *P* < 0.01).

## Results

### HPLC Analysis of LDOP Monosaccharide Composition

**Figure 1:**
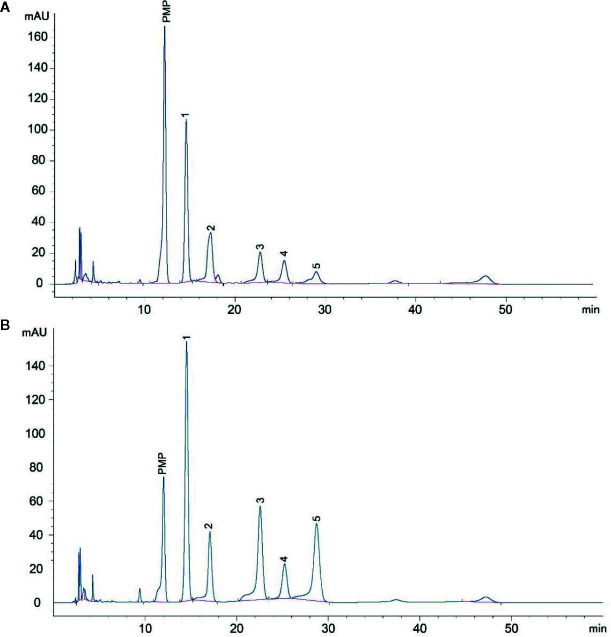
HPLC analysis of LDOP monosaccharide composition. **(A)** The composition of LDOP by HPLC analysis; 1: mannose, 2: galacturonic acid, 3: glucose, 4: galactose, 5: arabinose. **(B)** Standard monosaccharides. LDOP: The total polysaccharide from Dendrobium officinale Kimura & Migo.

LDOP was a Navajo white powder, which was primarily composed of total sugar (22.59%) and a small content of flavonoid (1.53%), total polyphenol (0.28%), protein (0.36%), and dendrobine (0.00078%). In addition, the monosaccharide composition of LDOP was determined by the HPLC method of precolumn derivatization with PMP. As shown in **Figure 1**, LDOP was a heteropolysaccharide comprising mannose, galacturonic acid, glucose, galactose, and arabinose at a molar ratio of 2.0:1.1:0.7:0.5:0.4. Next, we got LDOP-1 for subsequent experiments by our previous plan (Yang et al., 2020).

### LDOP-1 Mitigated Ethanol-Induced Mucosal Injury

**Figure 2:**
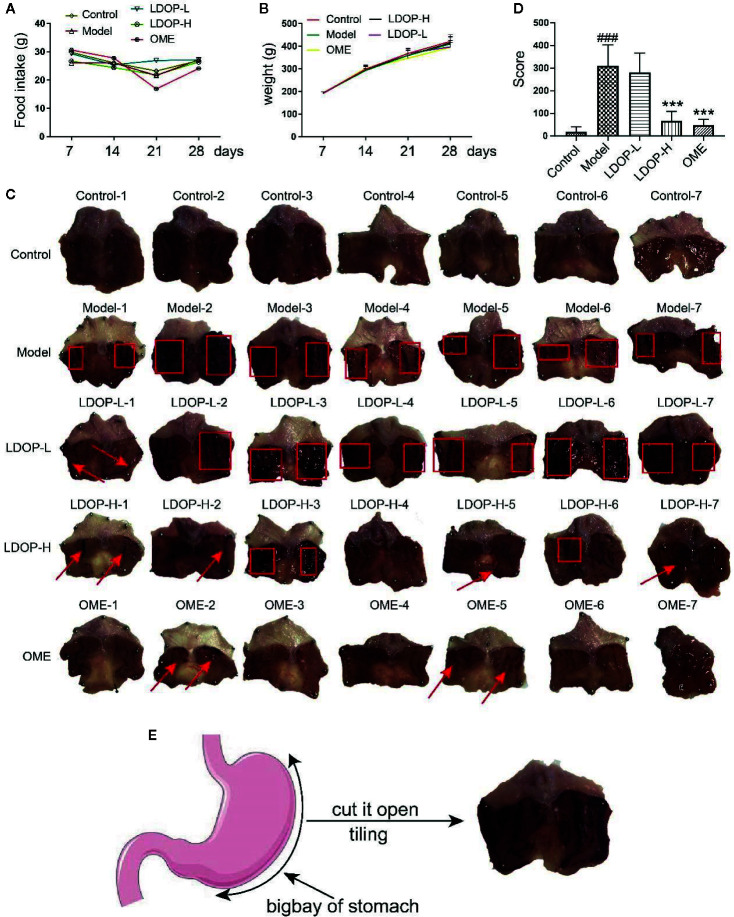
Effects of LDOP-1 on food intake and weight, as well as the macroscopic images of the gastric mucosal damage and injury score in the rat subjected to ethanol-induced gastric ulcer. Food intake **(A)** and weight **(B)** of rats were measured once a week. The images of gastric mucosal tissues **(C)** were quantification by the injury score, red rectangle stand for Bleeding zone, red arrow stand for Bleeding point. **(D)**. Data are expressed as the mean ± SD of three independent experiments. **(E)**. Pattern diagram of stomach anatomy. ^###^P < 0.001 compare the control group; ***P < 0.001 compare model group. LDOP-L stood for LDOP-1-L, LDOP-H stood for LDOP-1-H.

First, we measured the food intake and weight of rats once a week and found that LDOP-1 had no effects on each group of rats (**Figures 2A, B**). Then, we tested the gastric mucosal protection of LDOP-1 on the ethanol-induced gastric mucosal injury rat model (**Figure 2C**). The results showed that the model group, which was the oral administration of ethanol, induced many visible damages, including the glandular area hyperemia, linear hemorrhage necrosis, and the increasing mucosal edema. Moreover, the gastric mucosal injury score of the model group was significantly higher than that of the control group (**Figures 2D, E**). The pretreatment with low- or high-dose LDOP-1 and the OME group could significantly mitigate the mucosal injury area induced by ethanol and decrease the ulcer index in rat gastric mucosa compared with the model group.

### Histological Evaluation of Gastric Lesions

**Figure 3:**
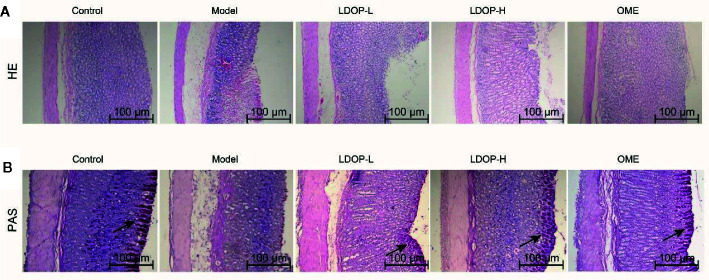
Effects of LDOP-1 on pretreatment on the histological evaluation of ethanol-induced gastric mucosal damage in rats by H&E staining and PAS staining. H&E staining **(A)** and PAS staining **(B)**, magnification (100×). LDOP-L stood for LDOP-1-L, LDOP-H stood for LDOP-1-H.

H&E staining of gastric tissues showed that the gastric mucosa of rats in the normal control group was smooth and flat, and the arrangement of basal epithelial cells was tight (**Figure 3A**). However, the model group evidently showed submucosal edema, epithelial cell separation, and shedding. In addition, the pathological injury of the gastric mucosa was reduced significantly by pretreating with Omeprazole and LDOP-1 compared with the model group.

PAS staining was used to assess the production of total glycoproteins, including mucins in the gastric epithelium (**Figure 3B**). Mucin in the gastric mucosa presented as the magenta in the PAS staining. As shown in **Figure 3B**, less mucin was observed in the model group than that observed in the normal control group. Evidently, LDOP-1 and Omeprazole upregulated the PAS staining intensity. Semi-quantitative analysis confirmed the results (**Supplementary Materials**).

### Effects of LDOP-1 on Oxidative Stress

**Figure 4:**
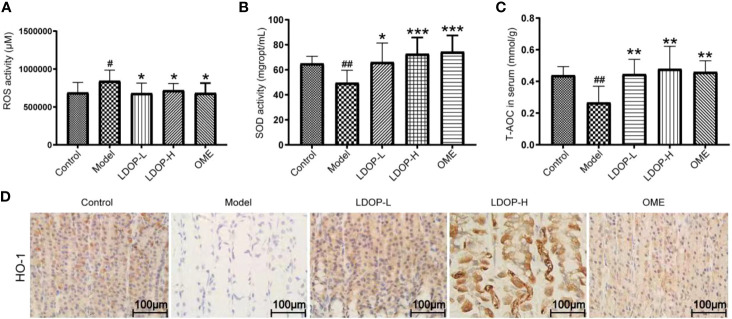
Effects of LDOP-1 on oxidative stress. ROS **(A)**, SOD **(B)** and T-AOC **(C)** were measured by biochemical Assays and the expression of HO-1 **(D)** was examined by immunohistochemical analysis (400×, brown yellow granules indicate positive reaction). Data are expressed as the mean ± SD of three independent experiments. ^#^P < 0.05, ^##^P < 0.01 compare the control group; *P < 0.05 and **P < 0.01, ***P < 0.001 compare model group. LDOP-L stood for LDOP-1-L, LDOP-H stood for LDOP-1-H.

Oxidative stress is a critical pathogenic factor during gastric ulceration, and we measured the expression level of ROS, SOD, T-AOC, and HO-1. ROS level in model group increased 1.22 times than normal control group, which was measured by fluorescent probe DCFH-DA. In addition, rats pretreated with either high-dose or low-dose LDOP-1 demonstrated a significant and dose-dependent reduction of ROS level decreased by 19.2% and 14.6% compared to the model group, respectively (**Figure 4A**). Lipid peroxidation in gastric ulcer was determined by measuring the SOD and T-AOC (**Figures 4B, C**). Compared with the control group, ethanol decreased the SOD bioactivity by 20.3% in the model group. T-AOC activity was also significantly down about 40.0% in the model group compared with that in the normal control group. In addition, the SOD and T-AOC production in high-dose LDOP-1 were increased prominently, which were successfully restored compared with the model group. The low-dose LDOP-1 and Omeprazole group showed similar results.

**Figure 5:**
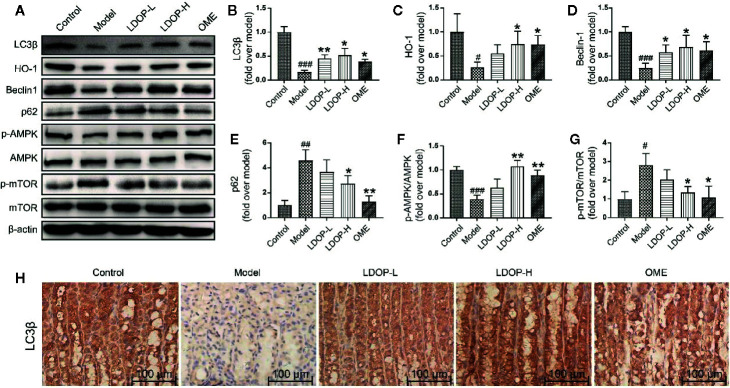
The representative western blot images of LC3β, HO-1, Beclin-1, p-AMPK, p62, p-mTOR and immunohistochemical image of LC3β showed that LDOP-1 induced autophagy via AMPK/mTOR signaling way in vivo. **(A)** The expression of LC3β, HO-1, Beclin-1, p-AMPK, p62, p-mTOR detected by Western blot. **(B-G)** Statistical analysis on LC3β, HO-1, Beclin-1, p-AMPK, p62, p-mTOR. Immunohistochemical image of LC3β **(H)** measured by immunohistochemical analysis (400×, brown yellow granules indicate positive reaction). Data are expressed as the mean  ±  SD of three independent experiments. ^#^P < 0.05, ^##^P < 0.01, ^###^P < 0.001 compare the control group; *P < 0.05 and **P < 0.01 compare model group. LDOP-L stood for LDOP-1-L, LDOP-H stood for LDOP-1-H.

Moreover, the expression level of HO-1 in the model group was less than that in the control group, but LDOP-1-L and LDOP-1-H were significantly upregulated compared with the model group (**Figure 4D**, **Figures 5A, C**).

### LDOP-1 Activated the Autophagy and Antioxidant Pathway in the Acute Alcohol-Induced Injury via the AMPK/mTOR Signaling Pathway

To determine whether LDOP-1 could induce autophagy, a number of autophagic markers were detected by Western blot and immunohistochemistry. As shown in **Figure 4**, the expression levels of LC3β, HO-1, Beclin-1, and p-AMPK in the model group were significantly decreased, and the expression levels of p62 and p-mTOR were upregulated compared with the control group. As such, pretreatment with LDOP-1 could activate the AMPK/mTOR signaling pathway, which could increase the expression level of LC3β, HO-1, Beclin-1, and p-AMPK and decrease the expression levels of p62 and p-mTOR (**Figures 5A–G**). Moreover, immunohistochemical staining showed that LC3β in the model group was poorly expressed compared with that in the control group, but pretreatment with LDOP-1 upregulated the expression level of LC3β, which was evidently better than the model group (**Figures 5A, B, H**).

### LDOP-1 Inhibited Apoptosis of Gastric Mucosal Cells

**Figure 6:**
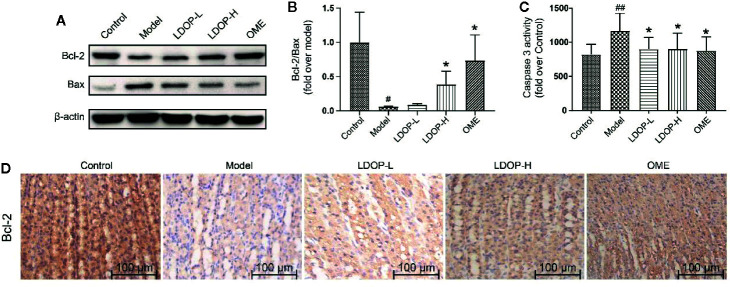
Effects of LDOP-1 on the expression of Bax, Bcl-2 and caspase 3 in vivo. The expression of Bax and Bcl_2_ were detected by Western blot **(A)**. Statistical analysis on Bcl-2/Bax **(B)**. The production of caspase 3 was measured by biochemical markers kit **(C)**. Immunohistochemical image of Bcl-2 **(D)** measured by immunohistochemical analysis (400×, brown yellow granules indicate positive reaction). Data are expressed as the mean ± SD of three independent experiments. ^#^P < 0.05, ^##^P < 0.01 compare the control group; *P < 0.05 compare model group. LDOP-L stood for LDOP-1-L, LDOP-H stood for LDOP-1-H.

Bcl-2 and Bax were a pair key of proteins that modulated apoptosis. In the model group, the expression level of proapoptotic protein Bax of gastric mucosal tissue significantly increased, and the antiapoptotic protein Bcl-2 was reduced compared with that in the control group. However, pretreatment with LDOP-1 significantly attenuated ethanol-induced changes of Bax and Bcl-2 (**Figures 6A, B, D**). During apoptosis, caspase 3 served as the initiator and effector. As such, the activity of caspase 3 in the model group dramatically increased compared with that in the normal control group, whereas pretreatment with LDOP-1 significantly decreased the expression level of caspase 3 compared with that in the model group (**Figures 6A, C**).

### LDOP-1 Protected GES-1 From Ethanol-Induced Cell Viability Loss

**Figure 7:**
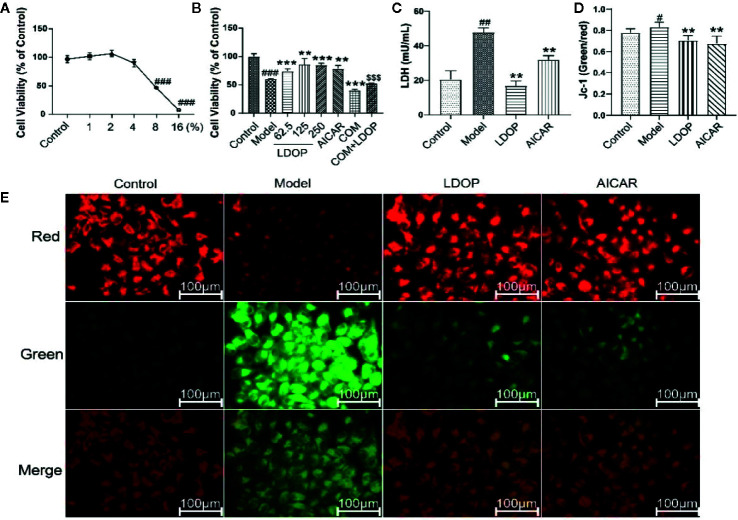
Effects of ethanol and LDOP-1 on GES-1 cells viability measured by MTT assay, LDH release and mitochondrial membrane potential. Effects of ethanol **(A)** and LDOP-1 **(B)** on GES-1 cells were measured by MTT assay. LDH release **(C)** was measured by LDH Cytotoxicity Assay Kit. Mitochondrial membrane potential was detected by JC-1 assay Kit **(D)**. Immunofluorescence image of JC-1 measured by immunofluorescence technique **(E)**. In the mitochondrial membrane potential is high, JC - 1 gathered in the mitochondrial matrix (matrix), the formation of polymer (J - aggregates), can produce red fluorescence; When the mitochondrial membrane potential was low, jc-1 could not accumulate in the matrix of the mitochondria. At this time, jc-1 was monomer and could produce green fluorescence. Data are expressed as the mean ± SD of three independent experiments. ^#^P < 0.05, ^##^P < 0.01, ^###^P<0.001 compare the control group; **P < 0.01, ***P < 0.001 compare model group, ^$$$^P < 0.001 compared with compound C group. LDOP stood for LDOP-1.

First, we filtered out the concentration of ethanol, which ranged from 1% to 16% and then used a different dose of LDOP-1 to investigate cell viability by MTT assay. As shown in **Figure 7A**, the data indicated that 8% ethanol could reduce cell viability to approximately 50%. Then, to determine whether LDOP-1 could protect gastric epithelial cell from ethanol-induced viability loss, GES-1 were pretreated with LDOP-1 (250, 125, 62.5 μg/mL), 0.5 mM AICAR, and 1 nM compound C (COM) before being incubated with ethanol at 8% concentration (v/v) for 2 h. The results showed that ethanol significantly decreased cell viability, which was 60% of the control group. Pretreatment with LDOP-1 at 250, 125, and 62.5 μg/mL remarkably diminished the decrease of cell viability compared with the model group. In addition, when pretreated with AICAR at 0.5 mM, which was the agonist of autophagy, the viability of GES-1 increased significantly compared with the control group. Moreover, when pretreated with the inhibitor autophagy, compound C suppressed cell viability, which was evidently lower than the model group, but GES-1 viability treated with compound C and LDOP-1 was evidently higher than that treated only with compound C (**Figure 7B**).

In this study, the reduction in LDH release was used to evaluate the protection effects of LDOP-1. The results indicated that ethanol treatment increased LDH release rapidly compared with the control group, but LDOP-1 effectively reduced the production of LDH to 24.3 ± 4.6, which proved that LDOP-1 could prevent GES-1 from apoptosis destructing the integrity of the cell membrane (**Figure 7C**).

To investigate the effect of LDOP-1 on the mitochondrial function, mitochondrial membrane potential (ΔΨm) was estimated by using the JC-1 probe, which was quantified by the fluorescence ratio of red to green. As shown in the control cells, red-polarized mitochondria and green-depolarized mitochondria were detected, which represented the ΔΨm under physiological conditions. However, red fluorescence was evidently weakened, and green fluorescence was enhanced in ethanol treatment; LDOP-1 and AICAR pretreatment for 12 h reversed the changes in fluorescence successfully (**Figure 7E**). Similarly, we also proved that the ratio of the model group was higher than that of the normal control group, and LDOP-1 pretreatment presented the protective effect compared with the model group (**Figure 7D**).

### LDOP-1 Enhanced GES-1 Antioxidant Ability

**Figure 8:**
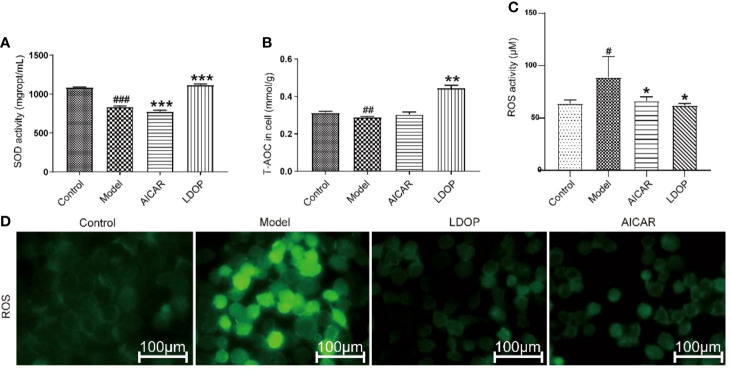
Effects of LDOP-1 on the production of SOD, T-AOC and ROS. **(A–C)** The levels of SOD, T-AOC and ROS were measured using a biochemical markers kit. The expression of ROS was detected by Immunofluorescence technique **(D)**. Data are expressed as the mean ± SD of three independent experiments. ^#^P < 0.05, ^##^P < 0.01, ^###^P < 0.001 compare the control group; *P < 0.05 and **P < 0.01, ***P < 0.001 compare model group. LDOP stood for LDOP-1.

The effect of LDOP-1 attenuated oxidative stress, which was a critical pathogenic factor, by measuring the expression level of SOD and T-AOC on GES-1. Compared with the control group, ethanol decreased the SOD level by 23.2% and T-AOC bioactivity by 58.5%, whereas LDOP-1 pretreatment enhanced the contents of SOD by 15.1%, T-AOC by 2.4 times (**Figures 8A, B**). To explore whether LDOP-1 could exert its protection via inhibiting the production of ROS, we assayed the generation of ROS with the non-fluorescent probe 2′,7′-dichlorofluorescin diacetate (DCFH-DA). GES-1 cells were treated with 8% ethanol for 3 hours after pretreated with LDOP-1 (250 μg/mL) for 12 hours. The results showed that 8% ethanol (v/v) increased the mean fluorescence intensity in the GES-1 compared with the control group. However, LDOP-1 and AICAR pretreatment decreased the fluorescence intensity significantly compared with the model group (**Figure 8D**). Similarly, the expression level of ROS in the model group was higher than the control group; LDOP-1 and AICAR pretreatment reverted the generation of ROS evidently compared with the model group by quantitative analysis (**Figure 8C**).

### Expression of LC3β in Relation to Time Effect and Dosage Effect

**Figure 9:**
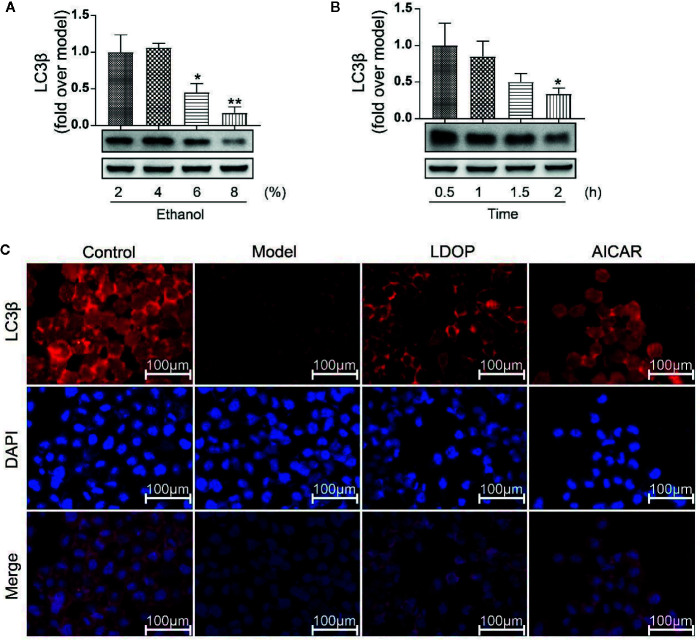
The expression of LC3β when treated with 2 %, 4 %, 6 %, 8 % concentration ethanol for 0.5 h, 1 h, 1.5 h, 2 h and effects of LDOP-1 on it. **(A)** The expression of LC3β at the ethanol at concentrations of 2 %, 4 %, 6 %, 8 %. **(B)** The expression of LC3β for 0.5 h, 1 h, 1.5 h. **(C)** Immunofluorescence image of LC3β measured by immunofluorescence technique (GES-1 were treated with 8% ethanol for 2 h after pretreatment with LDOP-1). Data are expressed as the mean ± SD of three independent experiments. *P < 0.05 and **P < 0.01 compare to 2 % concentrations ethanol or 0.5 h group. LDOP stood for LDOP-1.

LC3β was a key regulator of autophagy induction; thus, we detected the expression level of LC3β in relation to time effect and dosage effect. The results showed that GES-1 incubated with 6% and 8% ethanol for 2 h and significantly decreased the expression level of LC3β, but 4% ethanol had no statistical difference compared with 2% ethanol (**Figure 9A**). In another way, GES-1 were treated with 8% ethanol at 0.5, 1, 1.5, and 2 h. As shown in **Figure 9B**, the expression level of LC3β was decreased with dependence of time, and a significant difference was observed when treated for 2 h compared with that treated for 0.5 h. Therefore, GES-1 were treated with 8% ethanol for 2 h after pretreatment with LDOP-1 in subsequent experiments. The results indicated that the model group had lower reddish-blue fluorescent responses from LC3β than the control group. LDOP-1 and AICAR evidently improved reddish-blue fluorescence (**Figures 9A-C**).

### LDOP-1 Activated the mTOR/AMPK Signaling Pathway *in Vitro*

**Figure 10:**
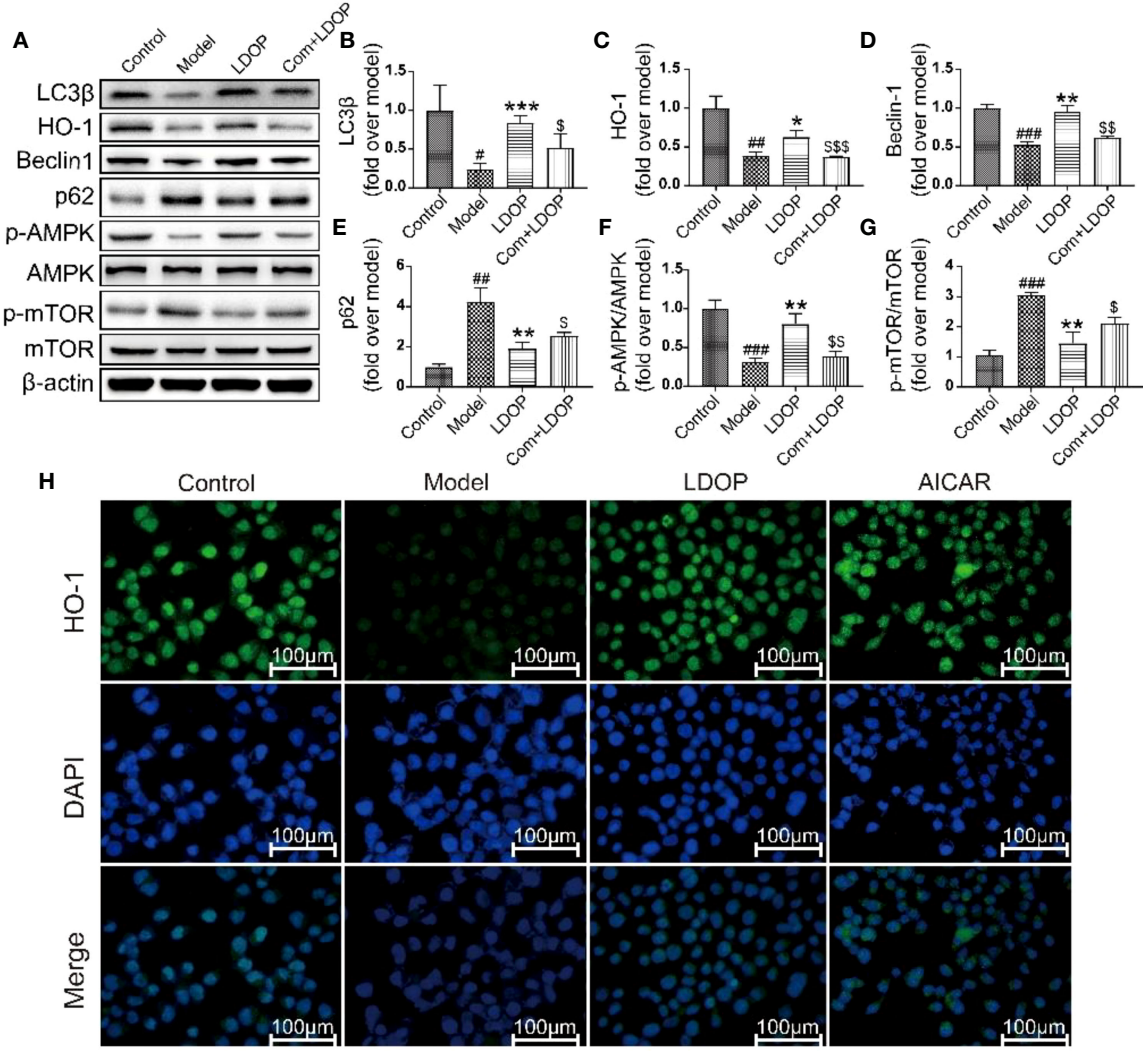
LDOP-1 could activate the autophagy by mTOR/AMPK signaling way in vitro. **(A)** The expression of LC3β, HO-1, Beclin-1, p-AMPK, p62, p-mTOR detected by Western blot. **(B-G)** Statistical analysis on LC3β, HO-1, Beclin-1, p-AMPK, p62, p-mTOR. Immunohistochemical image of HO-1 **(H)** measured by immunofluorescence technique. Data are expressed as the mean ± SD of three independent experiments. ^#^P < 0.05, ^##^P < 0.01, ^###^P < 0.001 compare the control group; *P < 0.05 and **P < 0.01, ***P < 0.001 compare model group, ^$^P < 0.05, ^$$^P < 0.01, ^$$$^P < 0.001 compared with LDOP-1 group. LDOP stood for LDOP-1.

To determine whether LDOP-1 activated the pathway of autophagy, GES-1 were treated with 8% ethanol for 2 h prior to Western blot analysis using antibodies against a number of autophagic markers (**Figure 10A**). As shown in **Figure 10A**, the expression levels of LC3β, HO-1, Beclin-1, and p-AMPK in the model group decreased, and the expression level of p62 and m-TOR significantly increased compared with that in the control group. By contrast, pretreatment with LDOP-1 evidently upregulated the expression level of LC3β, HO-1, Beclin-1, and p-AMPK and downregulated the expression level of p62 and m-TOR (**Figures 10B–G**). In addition, HO-1 was retained in GES-1 of the model group evaluated by immunofluorescence staining under a fluorescence microscope for the darker greenish-blue fluorescence. LDOP-1 treatment substantially improved the expression level of HO-1 by increasing in the intensity of greenish-blue staining (**Figures 10A, C, H**).

### LDOP-1 Inhibited Ethanol-Induced Apoptosis *in Vitro*

**Figure 11:**
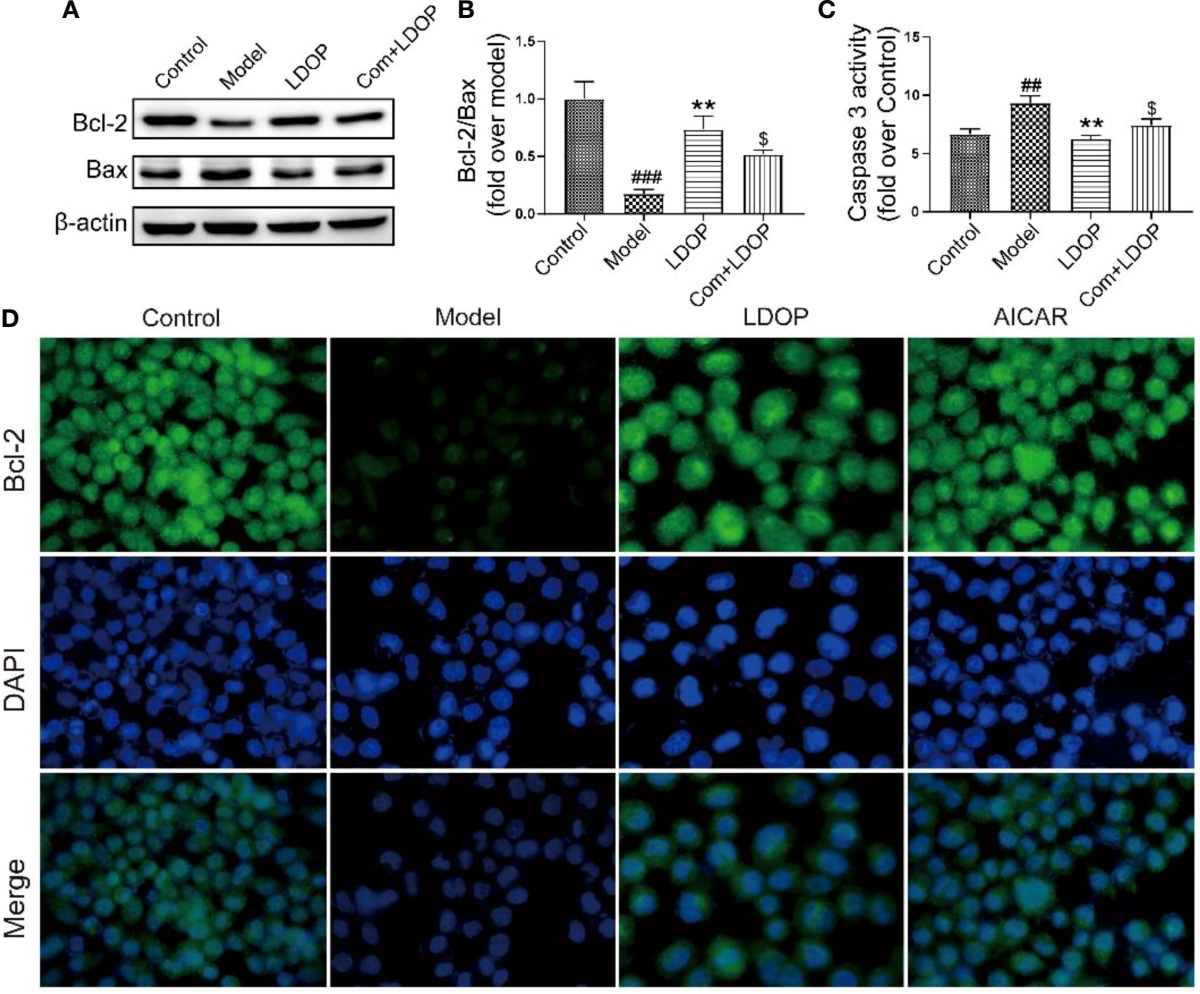
LDOP-1 inhibited the expression of Bax, caspase 3 and boost of Bcl-2. **(A)** The expression of Bax and Bcl_2_ detected by Western blot. **(B)** Statistical analysis on ratio of Bax to Bcl-2. **(C)** The production of caspase 3 detected by biochemical markers kit. **(D)** Immunohistochemical image of Bcl_2_ measured by immunofluorescence technique. Data are expressed as the mean ± SD of three independent experiments. ^##^P < 0.01, ^###^P<0.001 compare the control group; **P < 0.01 compare model group. ^$^P < 0.05 compare with LDOP-1 group. LDOP stood for LDOP-1.

Ethanol administration could trigger marked gastric apoptosis as demonstrated by the increase of proapoptotic signals, such as Bax, Caspase 3, and lower Bcl-2 compared with the control group. Notably, LDOP-1 and compound C with LDOP-1 counteracted these changes by inhibiting the caspase 3 and Bax protein expression compared with the model group with boosting of Bcl-2 (**Figures 11A-C**). In addition, the expression level of Bcl-2, detected by immunofluorescence, showed darker greenish-blue fluorescence compared with the control group. Meanwhile, LDOP-1 and AICAR restored the greenish-blue fluorescence (**Figures 11A, D**).

## Discussion

Alcohol is fat-soluble and excessive drinking caused the generation of acetaldehyde after acetaldehyde dehydrogenase (ADH) metabolism in the stomach. Then, it reduced the gastric mucosal injury by stimulating mucosal epithelial cell apoptosis and oxidative stress in gastric tissue (Zhang et al., 2019). This research found that absolute ethanol administration could cause glandular area hyperemia and linear hemorrhage necrosis, but LDOP-1 could reduce the damage. In addition, in vitro, GES-1 viability was reduced after 8% ethanol exposure for 2 h. Moreover, LDOP-1 reduced the release of LDH and increased mitochondrial membrane potential to prevent GES-1 from being damaged. We also found that *Dendrobium officinale* Kimura & Migo leaves (LDOP-1) reversed the Bax/Bcl_2_ ratio and the levels of caspase 3 in vivo and vitro, which indicated that LDOP-1 might be related to the decrease of gastric mucosal cell apoptosis. This finding was consistent with other research that polysaccharides from LDOP-1 had protective effects on gastric mucosal injury by downregulating the Bax/Bcl-2 ratio and caspase 3 activation in the gastric mucosa (Zeng et al., 2017).

Moreover, ethanol could diffuse across the cell membrane and generate ROS (Maity et al., 2009). In general, our body had the ability to clear ROS and keep it in balance but oxidative stress would be in an imbalance when the production of ROS beyond the body’s ability to neutralize them (Kwon et al., 2019). Consequently, as the second messenger, ROS changed the line plastochondria membrane potential, then caused in its butcher the environment changed to cause the cell death (Gonzalez et al., 2007; Kumari and Kakkar, 2012). Furthermore, the activity of SOD, T-AOC, and HO-1 was consumed by ethanol when damaging the gastric mucosa (Qin et al., 2019). In our study, LDOP-1 pretreatment evidently reduced the production of ROS and improved the expression level of SOD, T-AOC, and HO-1. Therefore, these findings indicated that LDOP-1 could protect ethanol-induced gastric mucosal injury probably involved in decreasing oxidative stress.

Autophagy played an important role in the protective effects of the gastric mucosa by inhibiting ethanol-induced ROS generation, depredating antioxidant enzymes, and lipid peroxidation, which could lead to oxidative stress (Chang et al., 2017). Thus, to determine whether autophagy was associated with the protection of LDOP-1 against ethanol-induced gastric mucosal injury in vivo and vitro, autophagy was assessed using Western blot and immunofluorescence technique. LC3β was a molecular marker of autophagy (Hou et al., 2019); thus, we first tested the expression level of LC3β at different times and concentrations of alcohol. The finding showed that LC3β had the lowest amount of expression level when GES-1 was incubated with 8% ethanol for 2 h, demonstrating the disappearance of autophagy. However, pretreatment with LDOP-1 increased the levels of LC3β successfully, indicating the activation of autophagy. Beclin1 was the mammalian ortholog of yeast Atg6, which had a central role in autophagy (Kang et al., 2011). Beclin1 increased after treatment with LDOP-1. The mammalian target of rapamycin (mTOR) was another key regulator of autophagy initiation, and activated mTOR could inhibit the occurrence of autophagy (Kim et al., 2011). AMPK played an important role in autophagy, serving as the upstream regulators of mTOR (Mihaylova and Shaw, 2011), and AMPK could be stimulated by ALCRA (Hunter et al., 2011). Similarly, recent studies showed that polysaccharide simultaneously increased AMPK and inhibited mTOR triggering autophagy-associated apoptosis (Medeiros et al., 2019; Liang et al., 2019). Our study showed that ethanol dramatically improved the expression level of p-mTOR and declined p-AMPK, but LDOP-1 upregulated p-AMPK and downregulated p-mTOR expression. Compound C, the inhibitor of AMPK (Emerling et al., 2007), had a lower expression level of p-AMPK compared with LDOP-1 pretreatment by working with LDOP-1 to pretreat GES-1. Accordingly, LDOP-1 could activate autophagy by the AMPK/mTOR signaling pathway to reduce the gastric mucosal injury in *vivo* and *vitro*.

LDOP-1 could reverse ethanol-induced gastric mucosal injury by reducing oxidative damage and activating autophagy through the upregulation of p-AMPK to inhibit the expression level of p-mTOR in *vivo* and *vitro*. This study also had inadequacy. Polysaccharides should be further separated and purified to determine the efficacy.

## Conclusion

**Figure 12:**
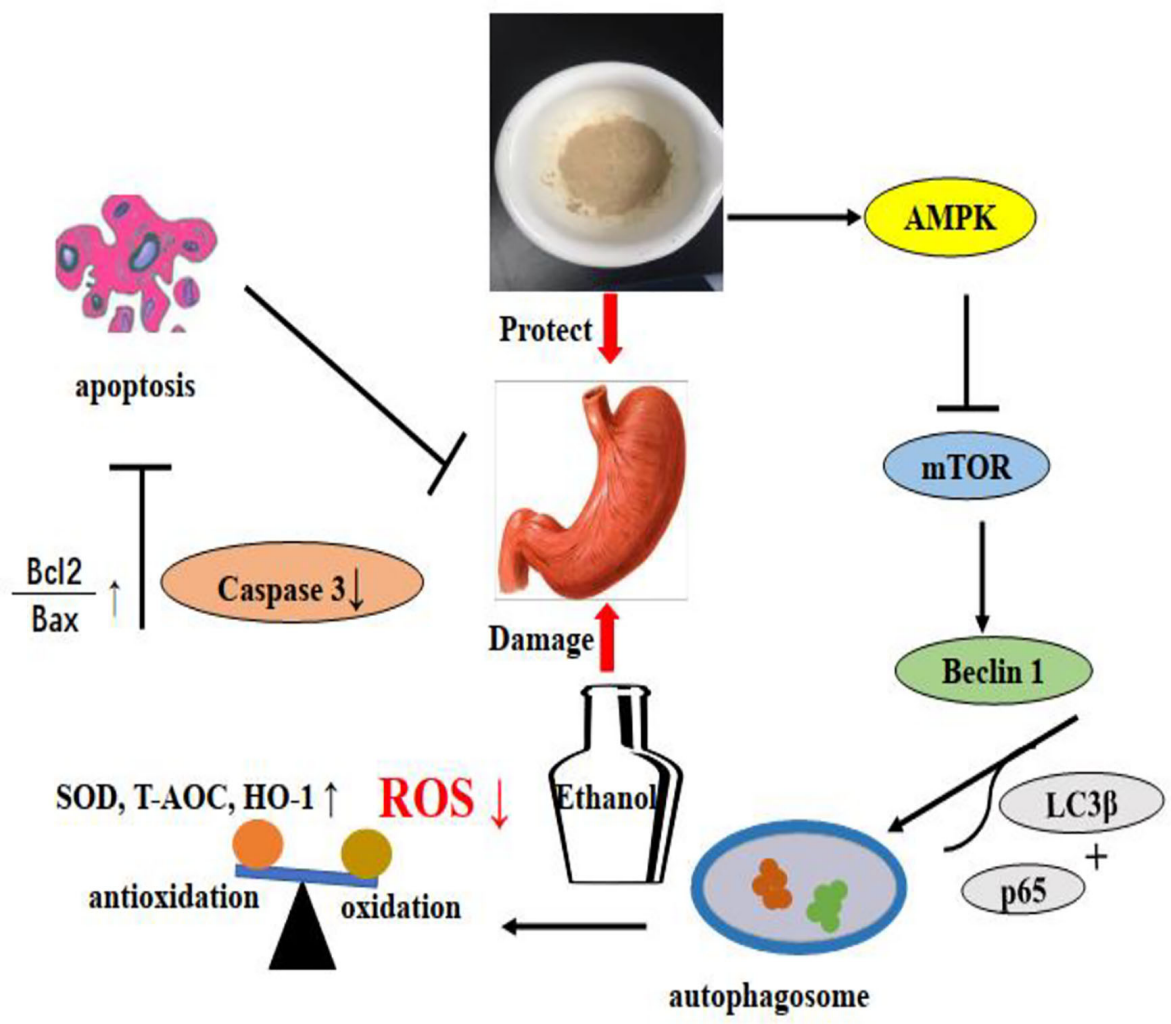
LDOP-1 protected against ethanol-induced gastric mucosal injury via AMPK/mTOR signaling pathway.

Based on previous findings, our research demonstrated for first time that polysaccharides from *Dendrobium officinale* Kimura & Migo leaves (LDOP-1) could alleviate ethanol-induced gastric mucosal injury in *vivo* and *vitro*. The effects might be mediated by certain antioxidants by significantly reducing the production of ROS and improving the levels of SOD, T-AOC, and HO-1. The underlying mechanism might be that LDOP-1 could activate the expression level of p-AMPK, LC3β, and Beclin 1; inhibit the level of p-mTOR and p62; increase the ratio of Bcl-2 and Bax; and decrease the activity of caspase 3, but these phenomena could be abolished by compound c. LDOP-1 might exert the gastroprotective effects by the AMPK/mTOR signaling pathway (**Figure 12**). Furthermore, the results suggested that LDOP-1 could be a better natural agent to develop healthy products for its treatment of ethanol-induced gastric mucosal injury, thereby realizing the development of new resources.

## Data Availability Statement

The datasets generated for this study can be found in the article.

## Ethics Statement

The animal study was reviewed and approved by Zhejiang University of Technology Laboratory Animal Center.

## Author Contributions

SC, GL, and YK designed the research. LZ and TL wrote the paper. LZ, CZ, and YD performed the experiment. All authors contributed to the article and approved the submitted version.

## Funding

This study was supported by the National Key Research and Development Program (No. 2017YFC1702200); The National Science Foundation of China (No. 81673638, 81874352, 81803761); Ten-thousand Talents Program of Zhejiang Province (ZJWR0102035); The Funding for Young Talents Project of Zhejiang University of Technology (No. GY18034148004); and China Postdoctoral Science Foundation (No. 2019M652144).

## Conflict of Interest

The authors declare that the research was conducted in the absence of any commercial or financial relationships that could be construed as a potential conflict of interest.
