# Corrigendum: Polysaccharides of *Dendrobium officinale* Kimura & Migo leaves protect against ethanol-induced gastric mucosal injury *via* the AMPK/mTOR signaling pathway *in vitro* and *vivo*

**DOI:** 10.3389/fphar.2022.948569

**Published:** 2022-08-15

**Authors:** Yang Ke, Lianghui Zhan, Tingting Lu, Cong Zhou, Xue Chen, Yingjie Dong, Guiyuan Lv, Suhong Chen

**Affiliations:** ^1^ Collaborative Innovation Center of Yangtze River Delta Region Green Pharmaceuticals, Zhejiang University of Technology, Hangzhou, Zhejiang, China; ^2^ College of Pharmaceutical Science, Zhejiang Chinese Medical University, Hangzhou, Zhejiang, China

**Keywords:** *Dendrobium officinale* Kimura & Migo leaves (LDOP), polysaccharides, alcohol, gastric injury, autophagy

In the original article, there was a mistake in Figures 4D, 8D, 10H, 11D as published. Due to the negligence of the author in the drawing process, the pictures are placed repeatedly. The corrected Figures 4, 8, 10, 11 appears below.

**FIGURE 4:**
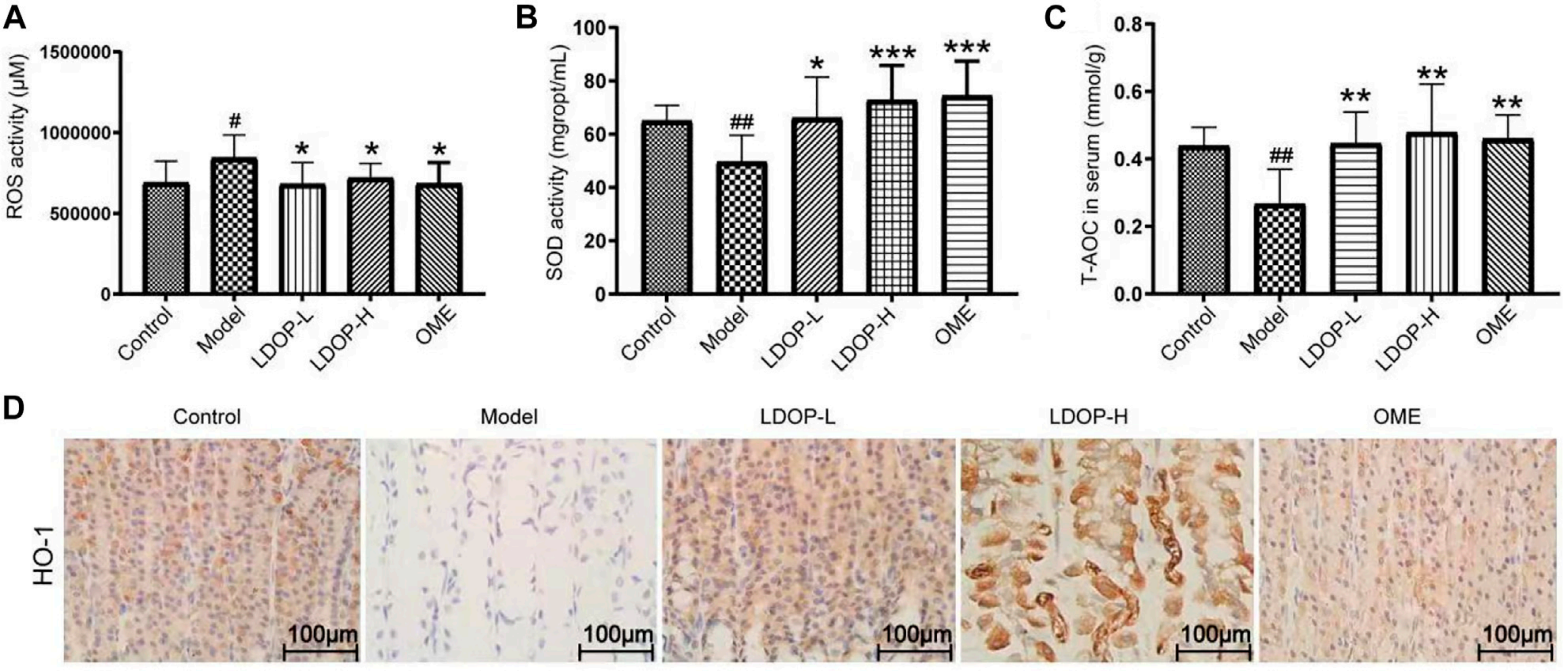
Effects of LDOP-1 on oxidative stress. ROS **(A)**, SOD **(B)** and T-AOC **(C)** were measured by biochemical Assays and the expression of HO-1 **(D)** was examined by immunohistochemical analysis (400×, brown yellow granules indicate positive reaction). Data are expressed as the mean ± SD of three independent experiments. #*p* < 0.05, ##*p* < 0.01 compare the control group; *p < 0.05 and **p < 0.01, ***p < 0.001 compare model group. LDOP-L stood for LDOP-1-L, LDOP-H stood for LDOP-1-H.

**FIGURE 8:**
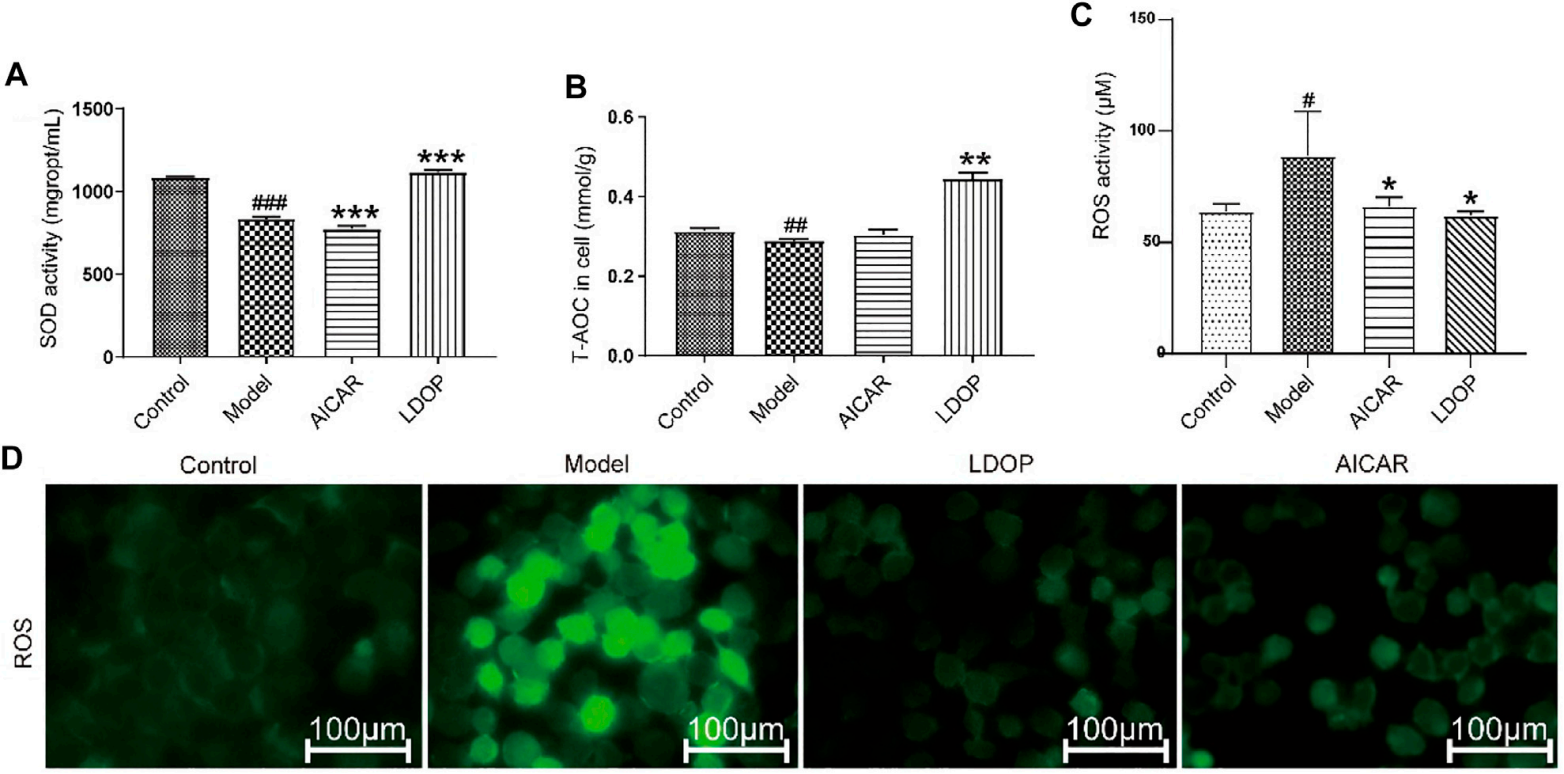
Effects of LDOP-1 on the production of SOD, T-AOC and ROS. **(A–C)** The levels of SOD, T-AOC and ROS were measured using a biochemical markers kit. The expression of ROS was detected by Immunofluorescence technique **(D)**. Data are expressed as the mean ± SD of three independent experiments. #*p* < 0.05, ##*p* < 0.01, ###*p* < 0.001 compare the control group; *p < 0.05 and **p < 0.01, ***p < 0.001 compare model group. LDOP stood for LDOP-1.

**FIGURE 10:**
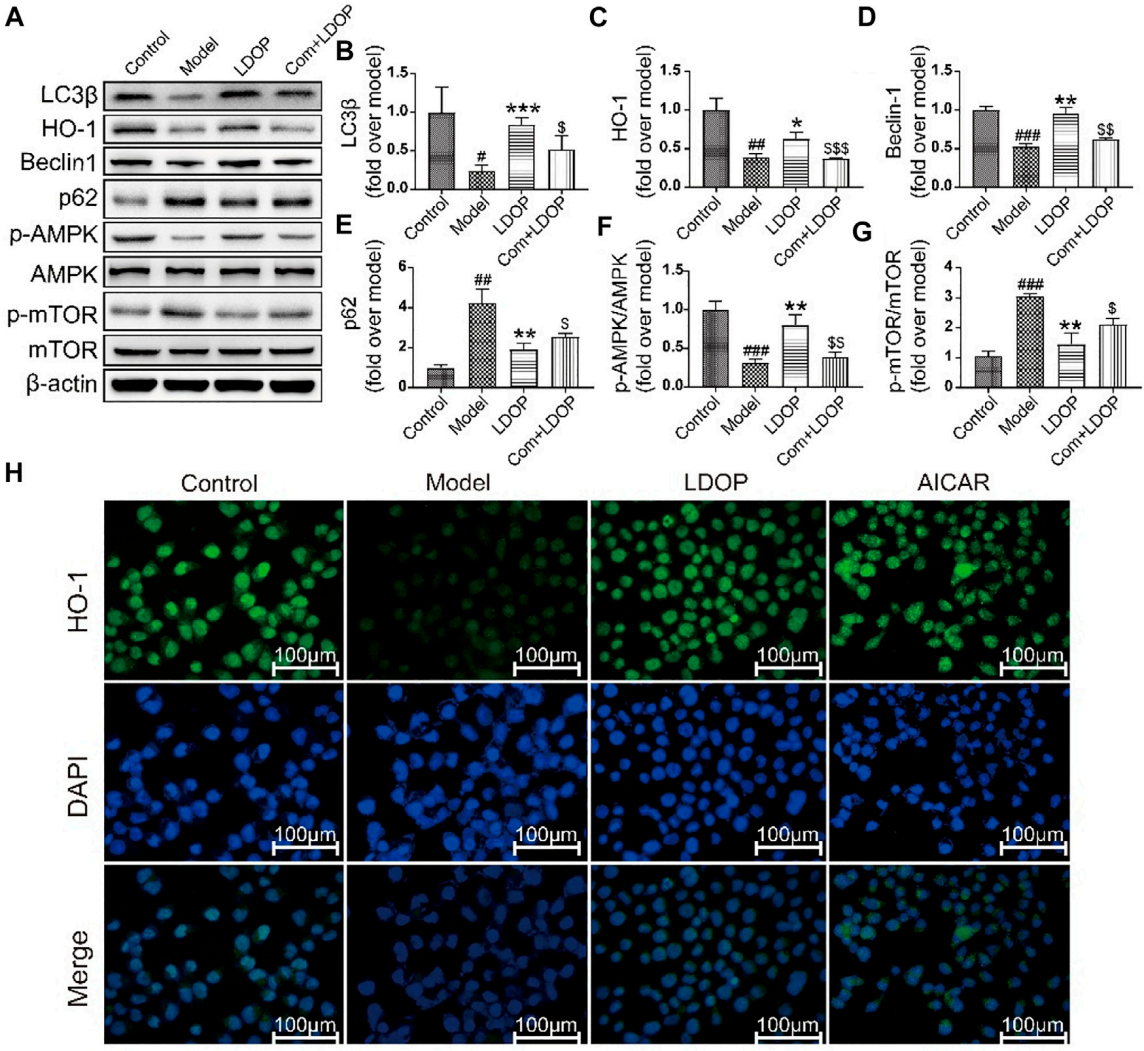
LDOP could activate the autophagy by mTOR/AMPK signaling way *in vitro*. **(A)** The expression of LC3β, HO-1, Becline-1, p-AMPK, p62, p-mTOR detected by Western blot. **(B–G)** Statistical analysis on LC3β, HO-1, Becline-1, p-AMPK, p62, p-mTOR. Immunohistochemical image of HO-1 **(H)** measured by immunofluorescence technique. Data are expressed as the mean ± SD of three independent experiments. ^#^ *p* < 0.05, ^##^ *p* < 0.01, ^###^ *p* < 0.001 compare the control group; *p < 0.05 and **p < 0.01, ***p < 0.001 compare model group, ^$^ *p* < 0.05, ^$$^ *p* < 0.01, ^$$$^ *p* < 0.001 compared with LDOP group.

**FIGURE 11:**
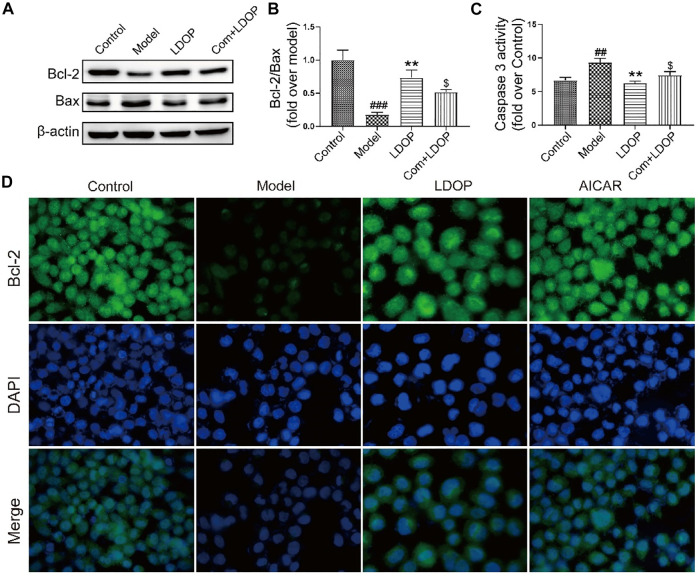
LDOP inhibited the expression of Bax, caspase 3 and boost of Bcl-2. **(A)** The expression of Bax and Bcl_2_ detected by Western blot. **(B)** Statistical analysis on ratio of Bax to Bcl-2. **(C)** The production of caspase 3 detected by biochemical markers kit. **(D)** Immunohistochemical image of Bcl_2_ measured by immunofluorescence technique. Data are expressed as the mean ± SD of three independent experiments. ^#^ *p* < 0.05, ^##^ *p* < 0.01, ^###^ *p* < 0.001 compare the control group; *p < 0.05 and **p < 0.01, ***p < 0.001 compare model group.

The authors apologize for this error and state that this does not change the scientific conclusions of the article in any way. The original article has been updated.

