# Supplementary figures and images for: Polysaccharides of Dendrobium officinale Kimura & Migo Leaves Protect Against Ethanol-Induced Gastric Mucosal Injury via the AMPK/mTOR Signaling Pathway in Vitro and vivo
Source: Front Pharmacol. 2020 Nov 11;11:526349. doi: 10.3389/fphar.2020.526349 (PMC7686799; doi:10.3389/fphar.2020.526349)

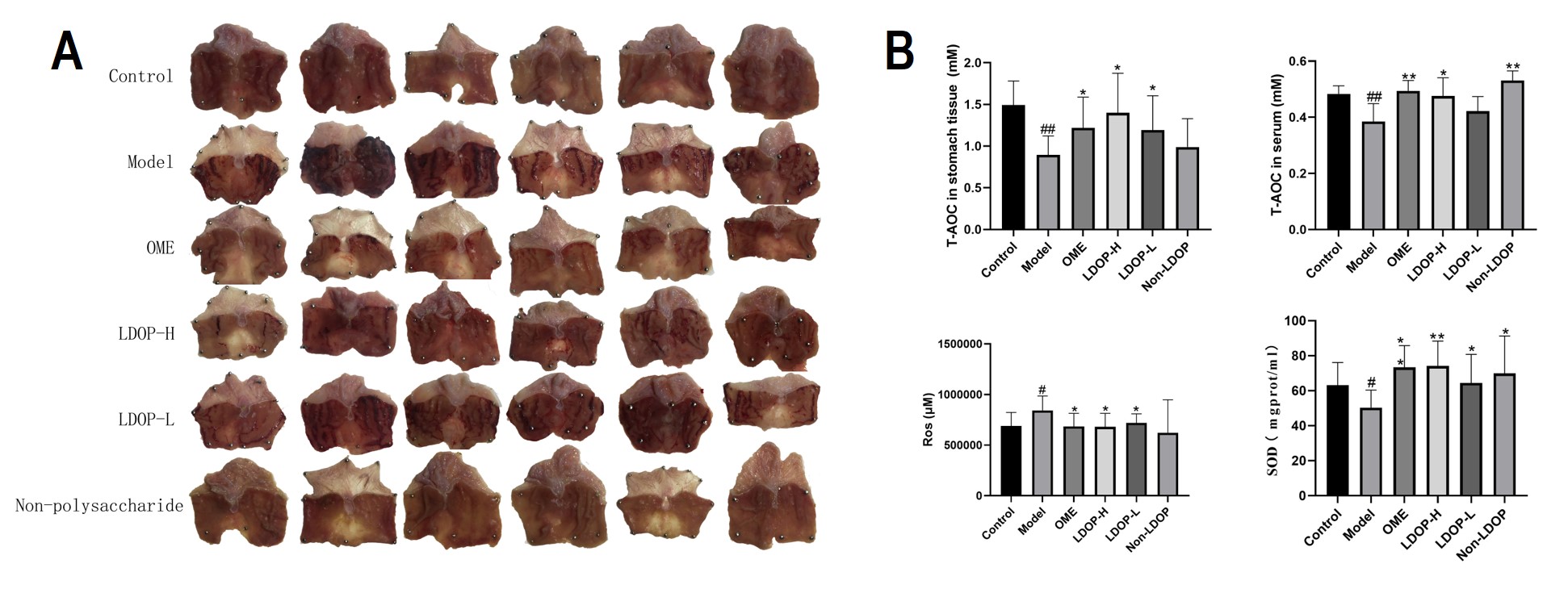

Supplement: Supplementary file 1 [file Image_1.jpeg]

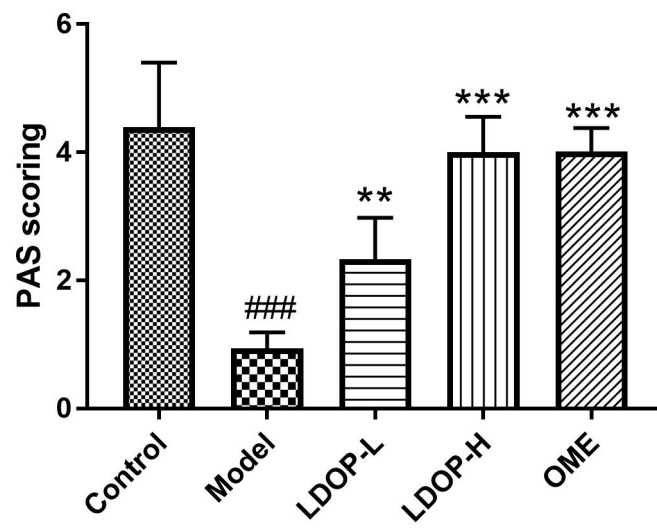

Scores from PAS. ###P<0.001 compare the control group; \*\*P<0.01 and \*\*\*P<0.001 compare model group

Supplement: Supplementary file 2 [file Image_2.pdf]
